# ELM—the eukaryotic linear motif resource in 2020

**DOI:** 10.1093/nar/gkz1030

**Published:** 2019-11-04

**Authors:** Manjeet Kumar, Marc Gouw, Sushama Michael, Hugo Sámano-Sánchez, Rita Pancsa, Juliana Glavina, Athina Diakogianni, Jesús Alvarado Valverde, Dayana Bukirova, Jelena Čalyševa, Nicolas Palopoli, Norman E Davey, Lucía B Chemes, Toby J Gibson

**Affiliations:** 1 Structural and Computational Biology Unit, European Molecular Biology Laboratory, Heidelberg 69117, Germany; 2 Collaboration for Joint PhD Degree between EMBL and Heidelberg University, Faculty of Biosciences; 3 Institute of Enzymology, Research Centre for Natural Sciences, Budapest 1117, Hungary; 4 Instituto de Investigaciones Biotecnológicas (IIBio) and Consejo Nacional de Investigaciones Científicas y Técnicas (CONICET), Universidad Nacional de San Martín. Av. 25 de Mayo y Francia, CP1650, Buenos Aires, Argentina; 5 Nazarbayev University, Nur-Sultan 010000, Kazakhstan; 6 Department of Science and Technology, Universidad Nacional de Quilmes - CONICET, Bernal B1876BXD, Buenos Aires, Argentina; 7 The Institute of Cancer Research, Chester Beatty Laboratories, 237 Fulham Rd, Chelsea, London SW3 6JB, UK

## Abstract

The eukaryotic linear motif (ELM) resource is a repository of manually curated experimentally validated short linear motifs (SLiMs). Since the initial release almost 20 years ago, ELM has become an indispensable resource for the molecular biology community for investigating functional regions in many proteins. In this update, we have added 21 novel motif classes, made major revisions to 12 motif classes and added >400 new instances mostly focused on DNA damage, the cytoskeleton, SH2-binding phosphotyrosine motifs and motif mimicry by pathogenic bacterial effector proteins. The current release of the ELM database contains 289 motif classes and 3523 individual protein motif instances manually curated from 3467 scientific publications. ELM is available at: http://elm.eu.org.

## INTRODUCTION

Short linear motifs (SLiMs), eukaryotic linear motifs (ELMs), MoRFs and miniMotifs, are a distinct class of protein interaction interface that is central to cell physiology (1,2). In the original 1990 definition, SLiMs were described as ‘linear, in the sense that 3D organization is not required to bring distant segments of the molecule together to make the recognizable unit.’ (3). This unexpected structural property was later explained by their frequent occurrence within intrinsically disordered regions of proteins or in exposed flexible loops within folded domains (1,4). The preference for flexible regions and their lack of tertiary structural constraints allows them to be accessible for protein–protein interaction and adopts the bound structure required for interaction with their binding partner.

The cell uses transient and reversible SLiM-mediated interactions to build dynamic complexes, control protein stability and direct proteins to the correct cellular compartment. Post-translational modification SLiMs act like switches that allow the transmission of cell state information to the wider protein population (5) and integrate different signaling inputs to allow decision-making on the protein level (6,7). Given the central regulatory role of SLiMs, they are now understood to be at the interface between biology and medicine. SLiMs are mutated in many human diseases including the degrons of tumor promoters in cancer (8,9) and are pervasively mimicked by pathogens through convergent evolution to hijack and deregulate host cellular functions (10–13). This understanding of the therapeutic relevance of SLiMs has resulted in an increased interest in drugging SLiM-mediated interactions (14).

Based on estimates obtained from high-throughput screening (HTS) experiments and computational studies, the human proteome is predicted to contain over 100 000 binding motifs and vastly more post-translational modification sites (PTMs) (4). However, motif discovery and characterization are hampered by computational and experimental difficulties (15) and only a small fraction of these anticipated sites have been discovered to date, which is underscored by the fact that we currently ignore the interaction partners for ∼75% of structural domain families (4). Because of the time consuming nature of literature curation, only a fraction of the experimentally discovered SLiM instances and classes are currently represented in the ELM resource. Therefore, improving the curation coverage of both known and novel motif classes is an important task for the the motif biology field.

The current census of SLiMs has been characterized over 30 years of small steps using cell biology and biophysical approaches. These advances are often limited by our inability to characterize SLiMs *in vivo* in the context of complex multiprotein assemblies and the difficulty of reproducing these assemblies *in vitro*. Nevertheless, the reductionist approach favored in motif biology has still resulted in numerous fundamental insights in cell biology. The application of medium and high-throughput approaches for the discovery of motifs, such as proteomic phage display (ProP-PD) (16) and peptides attached to Microspheres with Ratiometric Barcode Lanthanide Encoding (MRBLE-pep) (17), is now on the cusp of revolutionizing the field of motif biology. Consequently, a large body of motif data is on the verge of becoming available.

The ELM resource has an important role in guiding the development of these novel experimental approaches, as it is the only existing resource where motif definitions are described in the context of the underlying biology and evolution. SLiM curation remains the gold standard for motif data and the ELM instances will provide benchmarking data for these novel approaches and help define discriminatory motif attributes that will drive the discovery of novel motifs. This is in addition to the existing roles of the ELM resource in the molecular biology community as a repository of motif information, a server for exploring candidate motifs in protein sequences and a source of training data for bioinformatics tool development. As the 20th anniversary of ELM approaches, the resource remains a foundational hub for the motif community, and new tools such as *articles.ELM* (http://slim.icr.ac.uk/articles/) have been developed to assist the curation process in the face of the increased data that will become available in the near future.

## THE ELM RESOURCE

**Figure 1.**
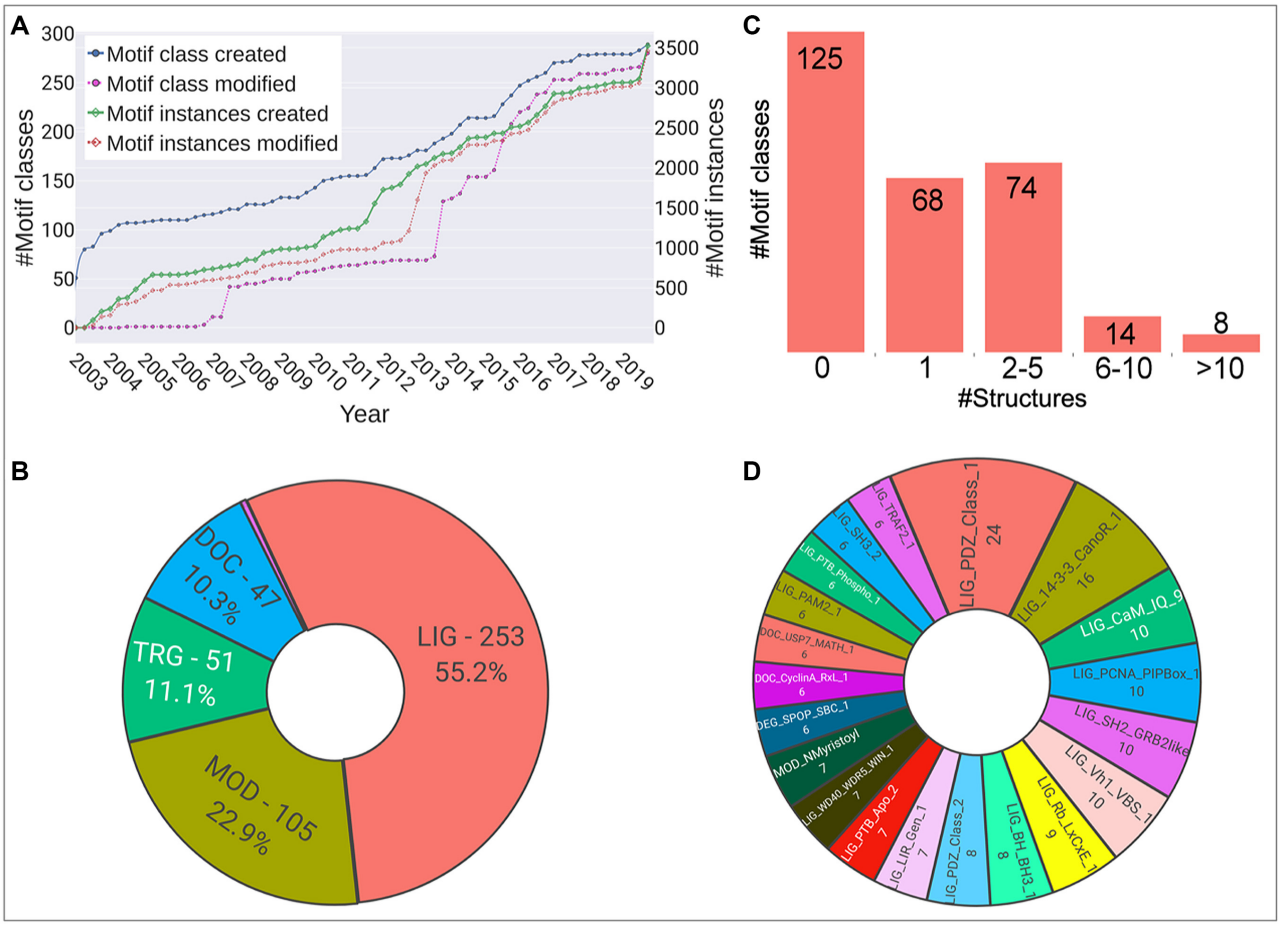
(**A**) Progression of the motif classes and instances integrated in the ELM resource. (**B**) Pie-chart showing count and proportion of new instance addition from each motif class type in the current ELM release. (**C**) Barplot showing the motif classes grouped according to the coverage of their instances by PDB structures, only one structure per instance has been considered for showing the coverage. In total, 164 ELM classes are covered by at least one structure. (**D**) Top 20 motif classes in terms of the number of representative PDB structures are shown. The plots were generated using plotly chart studio (https://chartstudio.plot.ly).

The ELM resource (http://www.elm.eu.org) contains two services: the ELM server for exploring candidate motifs and, the main focus of the current update, the ELM database. The ELM relational database is a repository that collects, classifies and curates experimental information on SLiMs. The ELM database has been under development for almost 20 years and has shown steady growth in the number of curated articles, collected motif instances and motif class definitions (18–23) (Figure 1). The ELM database classifies motif instances into class entries based on shared function, specificity determinants or binding partner. For each motif class, ELM provides a comprehensive report analogous to a short review describing the motif’s function, interacting domains, binding determinants and taxonomic range. Related motif classes such as those interacting with the same protein domain are grouped under a unique functional site class. Motif classes are also grouped by type based on their high level function as ligand (LIG), targeting (TRG), docking (DOC), degradation (DEG), modification (MOD) or cleavage (CLV) motifs. Each ELM motif class entry also provides a list of experimentally validated motif instances manually curated from the literature. For each instance, ELM curates the binding peptide (mapped to the protein entry in UniProt (24), the protein information, the relevant publication, the methods used to characterize the motif and information on the binding partner(s). If available, the binding affinity (typically as dissociation constants) and structural information are also collected. With the current release, ELM encompasses 3523 motif instances, 289 motif classes, 516 structures containing SLiM peptides and 3467 scientific publications. Table 1 provides a breakdown of the main data types in the ELM resource.

**Table 1.** Overview of the data stored in the ELM database

Functional sites		ELM classes		ELM instances		GO terms		PDB structures	ELM instances with affinity values	PubMed Links
Total	176		289		3523		791	516	265	3467
By category		LIG	163	Human	2090	Biological process	430			
		MOD	37	Mouse	341					
		DOC	31	Rat	150	Cellular component	163			
		DEG	25	Yeast	110					
		TRG	22	Fly	98	Molecular function	198			
		CLV	11	Others	734					

Programmatic access to the ELM resource is available through the REST API (for instructions see http://elm.eu.org/api/manual.html). For example, motif matches for the human p53 protein (UniProt accession:P04637) can be retrieved using the REST request http://elm.eu.org/start_search/P04637.tsv. Other features of the ELM resource have been outlined in the 2018 ELM paper (23) or earlier.

ELM motif data will become linked from PDBe-KB (25) and structures in ELM are now linked to PDBe (26) from the ELM structure page (http://elm.eu.org/pdbs/).

## NOVEL AND UPDATED ELM CLASSES

As novel aspects of motif biology have appeared, the ELM resource has at times changed curation focus to populate high profile or underpopulated biological pathways. In previous releases, this has included curation drives for SLiMs in viral proteins, conditionally regulated motif switches and motifs regulating cell cycle progression. The current release of ELM has continued this approach by focusing curation on DNA damage, the cytoskeleton, kinase specificity, SH2 domains and mimicry by pathogenic effectors. The current ELM release includes 21 new classes (Table 2), >400 new instances and 67 added structures. In addition, 12 existing motif classes have been updated to reflect advances in our understanding of those motifs (Table 2).

**Table 2.** Novel and revised ELM classes since the last ELM publication

Novel ELM classes		
ELM class identifier	Number of instances	ELM class (short) description
LIG_SH2_CRK	34	CRK family SH2 domain binding motif
LIG_PDZ_Wminus1_1	27	The C-terminal Trp-1 PDZ-binding motif is represented by a pattern like W(ACGILV)$.
LIG_SH2_STAP1	22	STAP1 Src Homology 2 (SH2) domain Class 2 binding motif
LIG_SH2_NCK_1	17	NCK Src Homology 2 (SH2) domain binding motif
LIG_PROFILIN_1	16	The polyproline profilin-binding motif is found in regulators of actin cytoskeleton.
LIG_PCNA_yPIPBox_3	12	The PCNA binding motifs include the PIP Box, PIP degron and the APIM motif, and are found in proteins involved in DNA replication, repair, methylation and cell cycle control. This is the variant for the yeast PIPbox.
LIG_REV1ctd_RIR_1	10	Several DNA repair proteins interact with the C-terminal domain of the Rev1 translesion synthesis scaffold through the Rev1-Interacting Region RIR motif that is centered around two neighboring Phe residues.
LIG_IBAR_NPY_1	7	A short NPY motif present in the bacterial effector protein Tir binds the I-BAR domain and is involved in actin polymerization.
LIG_MLH1_MIPbox_1	6	Proteins involved in DNA repair and replication employ conserved MIP-box motifs to bind the C-terminal domain of mismatch repair protein MLH1.
LIG_FXI_DFP_1	5	The DFP motif enables binding to the 2nd apple domain of coagulation factor XI (FXI) and plasma kallikrein heavy chain.
LIG_deltaCOP1_diTrp_1	5	Tryptophan-based motifs enable targeting of the tethering and (dis)assembly factors to the C-terminal mu homology domain (MHD) of the coatomer subunit delta, delta-COP.
LIG_CaM_NSCaTE_8	3	Short motif recognized by CaM that is only present in the Cav1.2 and Cav1.3 L-type calcium channels.
LIG_ARL_BART_1	2	The ligand motif present in N-terminus region of ARL2 and ARL3 proteins ensures GTD-dependent binding to BART and BARTL1.
LIG_PCNA_APIM_2	2	The PCNA-binding APIM motif is found in proteins involved in DNA repair and cell cycle control.
MOD_PRMT_GGRGG_1	24	A GGRGG motif recognized by the arginine methyltransferase for arginine methylation.
MOD_DYRK1A_RPxSP_1	22	Serine/Threonine residue phosphorylated by Arginine and Proline directed DYRK1A kinase.
DOC_PP4_FxxP_1	15	The FxxP-like docking motif recognized by the EVH1 domains of the PPP4R3 regulatory subunits of the PP4 holoenzyme.
DOC_PP4_MxPP_1	2	The MxPP-like docking motif recognized by the EVH1 domains of the PPP4R3 regulatory subunits of the PP4 holoenzyme.
DOC_MAPK_GRA24_9	2	A kinase docking motif that mediates interaction toward the ERK1/2 and p38 subfamilies of MAP kinases.
TRG_Pf-PMV_PEXEL_1	24	Plasmodium Export Element, PEXEL, is a trafficking signal for protein cleavage by PMV protease and export from Plasmodium parasites to infected host cells.
TRG_ER_FFAT_2	7	A variant of the classic MSP-domain binding FFAT (diphenylalanine [FF] in an Acidic Tract) motif.
ELM Classes with major revisions		
LIG_CaM_IQ_9	75	Helical peptide motif responsible for Ca^2+^-independent binding of the CaM.
LIG_SH2_GRB2like	35	GRB2-like Src Homology 2 (SH2) domain binding motif.
LIG_LIR_Gen_1	21	Canonical LIR motif that binds to Atg8 protein family members to mediate processes involved in autophagy.
LIG_PCNA_PIPBox_1	19	The PCNA-binding PIP Box motif is found in proteins involved in DNA repair and cell cycle control.
LIG_Vh1_VBS_1	15	An amphipathic α-helix recognized by the head domain of vinculin that is required for vinculin activation and actin filament attachment.
LIG_IRF3_LxIS_1	7	A binding site for IRF-3 protein present in various innate adaptor proteins and the viral protein NSP1 to trigger the innate immune responsive pathways.
MOD_CK2_1	34	Casein kinase 2 (CK2) phosphorylation site.
MOD_CK1_1	27	CK1 phosphorylation site.
MOD_CDK_SPxK_1	26	Canonical version of the CDK phosphorylation site that shows specificity toward a lysine/arginine residue at the [ST]+3 position.
MOD_CAAXbox	17	Generic CAAX box prenylation motif.
DOC_CyclinA_RxL_1	28	This motif is mainly based on cyclin A binding peptides and may not apply to all cyclins.
TRG_ER_FFAT_1	29	MSP-domain binding FFAT (diphenylalanine [FF] in an Acidic Tract) motif.

### DNA damage and repair

**Figure 2.**
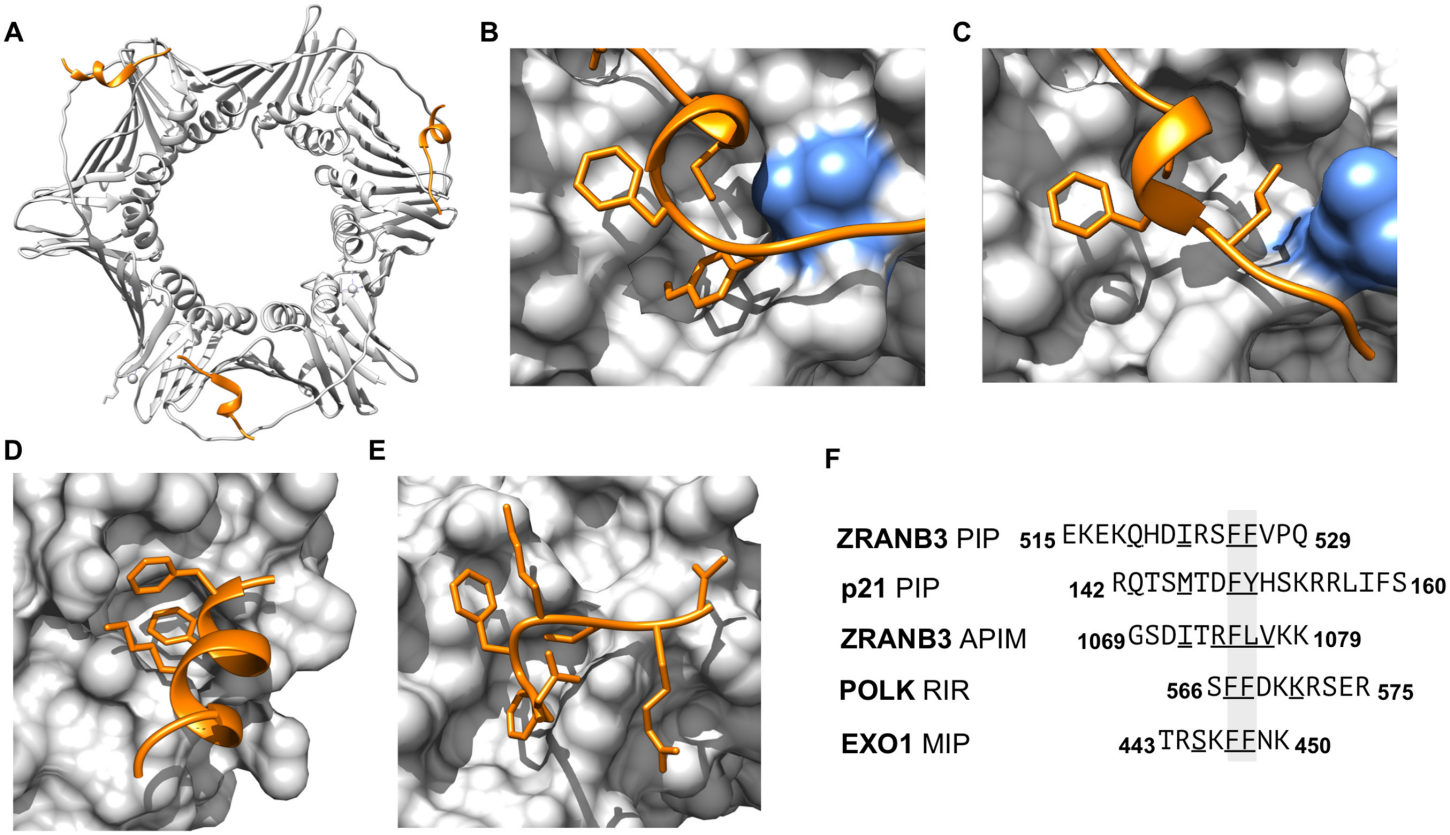
Structural information on representative DNA damage and repair motif instances and classes added in the current ELM update. (**A**) Structure of PCNA trimer in complex with PIP box of ZRANB3 [PDB ID: 5MLO] (77). (**B**) Closeup of the structure of PCNA PIP-binding pocket in complex with the PIP box of p21 [PDB ID: 1AXC] (34). (**C**) Close-up of the structure of PCNA PIP-binding pocket in complex with the APIM of ZRANB3 [PDB ID: 5YD8] (33). The blue residue in panels (B) and (C) shows the rearrangement of a leucine 126 in the PIP-binding pocket to accommodate the APIM peptide. (**D**) Close-up of the structure of the Rev1 C-terminal domain with the RIR motif of DNA polymerase kappa [PDB ID: 4FJO] (78). (**E**) Close-up of the structure of the C-terminal domain of the yeast MUTL alpha (MLH1/PMS1) bound to MIP box motif of Exo1 [PBD ID: 4FMO] (79). (**F**) Peptides from the structures of panels (A–E) aligned around their core hydrophobic residues. Underlined residues define the motif consensus residues in the peptide. Structural figures were prepared using the UCSF Chimera software (80).

In the new release of ELM, we have expanded our encoding of DNA damage and DNA repair motifs, providing a comprehensive picture of this large and diverse motif group (Figure 2). We have included several novel classes of proliferating cell nuclear antigen (PCNA)-interacting protein (PIP) box-like motifs including the APIM, and the related RIR and MIP motifs. We have expanded the definition of the PIP Box motif creating two classes that reflect the variation observed in metazoan versus fungal motifs. A variant motif representing the translesion synthesis polymerases is in preparation. The inclusion of the novel PIP-like motif classes has led to addition of 2 APIM (New class: LIG_PCNA_APIM_2), 10 RIR (New class: LIG_REV1ctd_RIR_1) and 6 MIP (New class: LIG_MLH1_MIPbox_1) motif instances. In addition, we updated the metazoan PIP Box (LIG_PCNA_PIPBox_1) with 19 instances and the fungal PIP Box (New class: LIG_PCNA_yPIPBox_3) with 12 instances. In total, the PIP-like motif classes have been expanded with 49 novel instances and 24 additional structures.

The accurate replication of DNA is essential for genome stability and for the faithful transmission of genetic information from mother to daughter cells. Successful DNA replication depends on the DNA synthesis machinery and on the efficient sensing of DNA damage in order to initiate the repair of DNA lesions or activate tolerance mechanisms that allow the replicative bypass of damaged DNA. The ability of cells to tolerate DNA damage is a key determinant of cancer therapy response, making DNA repair and damage proteins attractive drug candidates (27). PCNA, Mlh1 and Rev1 are hubs of genome maintenance networks responsible for the sensing and integration of DNA replication stress signaling. Protein partners interact with these hubs via PIP Box, MIP Box and RIR motifs, respectively.

Several DNA replication and repair pathways cooperate to ensure the reliable repair of different DNA damage types. The Mlh1 protein acts as a major signal integrator of the mismatch repair pathway. Partners from other repair pathways communicate with Mlh1 through the widely conserved MIP box motif (New class: LIG_MLH1_MIPbox_1) (28). The replicative bypass of DNA lesions is performed in a process termed translesion synthesis (TLS). Here, the Rev1 protein acts as a major scaffold that orchestrates the exchange of different polymerases. Rev1 is well suited for this job, because it can simultaneously bind Polζ and other TLS polymerases that have Rev1-interacting regions, so called RIR motifs (New class: LIG_REV1ctd_RIR_1) (29,30).

The PCNA protein is the ‘sliding clamp’ that encircles DNA at the replication fork. PCNA acts as a major scaffolding protein that orchestrates the assembly of replicative DNA polymerases, and integrates DNA damage signaling with tolerance mechanisms, working in combination with Rev1 to facilitate the recruitment of low-fidelity TLS polymerases to stalled replication forks and allow the replicative bypass of DNA lesions (31). The metazoan and fungal PIP Box (LIG_PCNA_PIPBox_1 and New class: LIG_PCNA_yPIPBox_3) (31) and APIM motifs (New class: LIG_PCNA_APIM_2) (32,33) mediate binding of a large number of PCNA-interacting proteins to the PCNA PIP Box cleft, including p21 and the Polη TLS polymerase. The Polι and Polκ TLS polymerases use a variant PIP-like motif that binds to the same binding cleft in PCNA (34,35). DNA Damage and cell cycle signaling are integrated by the p21 cyclin-dependent kinase inhibitor, which binds PCNA through its PIP Box and mediates cell cycle arrest in response to DNA damage to prevent cell cycle progress until replication can resume.

PIP-like motifs share a core hydrophobic helix that often contains a double-aromatic residue pair (36) (Figure 2), and several studies suggest that many PIP-like motifs are able to interact with at least two of these hub proteins (37,38). The available motif instances reveal the diversity but also the high conservation of PIP-like motifs, and point to the existence of a broader group of functionally and structurally related DNA damage and repair motifs that might show an unexpected degree of cross-functionality (37,38).

### Motif mimicry in bacterial effector proteins

A major ELM focus continuing from the last release has been the curation of the available literature on human motif mimicry by bacterial effector proteins. This curation drive mirrors a previous ELM release where the curation of the complete corpus of viral motif literature added over 200 novel ELM instances in 84 different viral taxa (10,20). Pathogens have an intimate relationship with their host and often produce proteins that mimic higher eukaryotic SLiMs to hijack, deregulate or rewire host pathways. This mimicry is facilitated by the ease of *ex nihilo* motif evolution due to the degeneracy of motifs and the rapid evolution of most bacterial and viral pathogens (1,39). The available literature on bacterial motifs is not as extensive as the viral motif literature but interest in the research field is increasing. ELM now contains information on >110 bacterial motif instances from 28 bacterial species mapping to 31 ELM classes. Our focus on bacterial mimicry has required us to improve ELM annotation for several topics, notably for cytoskeleton and membrane regulation, and for SH2 domain-binding motifs because ELM lacked entries that matched some of the effector motifs. For example, enteropathogenic *Escherichia coli* (EPEC) Tir protein is tyrosine-phosphorylated and then binds to the NCK SH2 domain (40). An NCK SH2 motif class entry has now been added to ELM (discussed below). The bacterial effector annotation in ELM is now close to being comprehensive with the current literature. It is clear that motif mimicry is a common feature of bacterial effector proteins.

**Figure 3.**
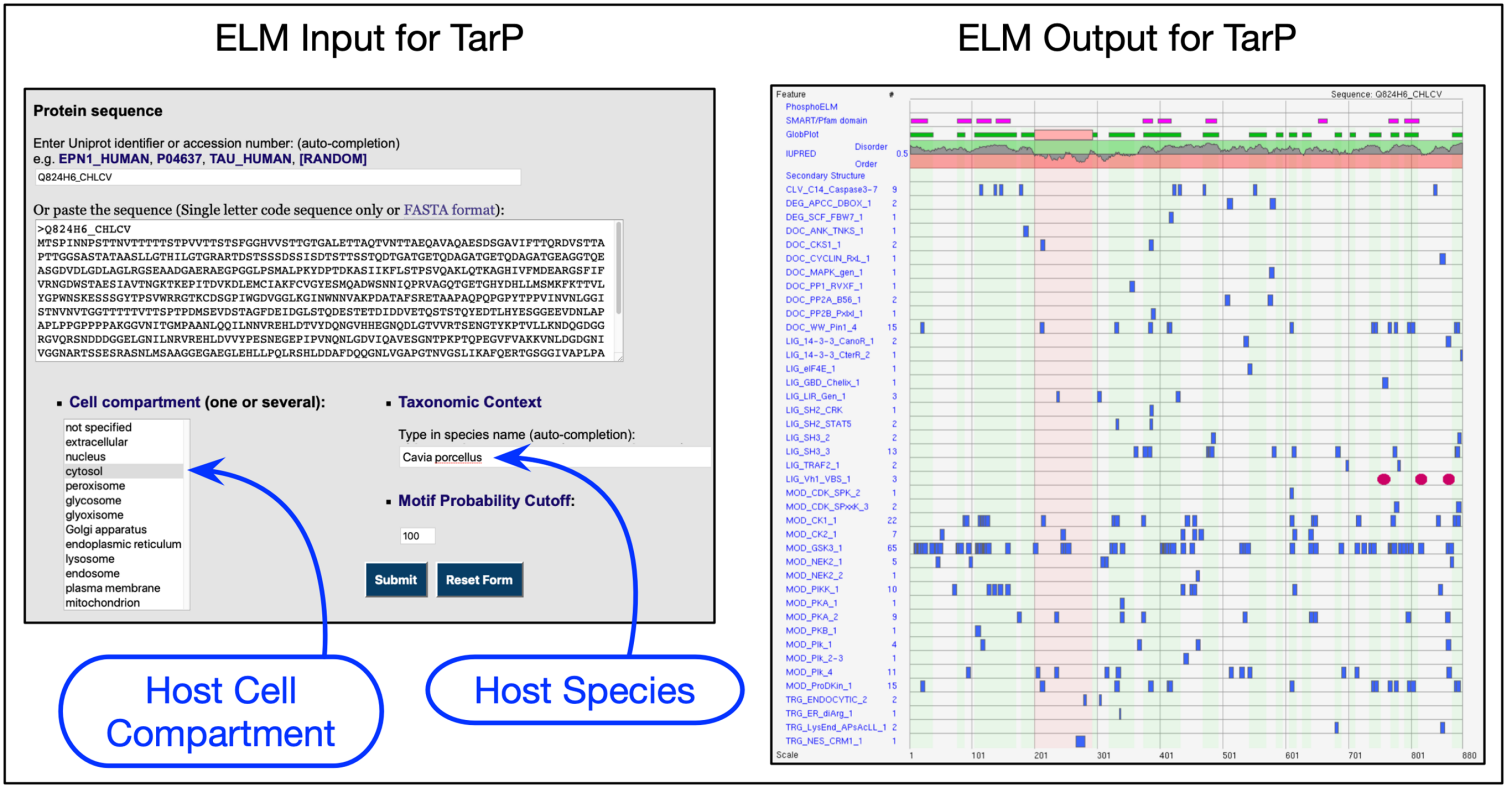
Setting up the ELM server correctly to query bacterial effectors for SLiM candidates using, as an example, the IDP-rich TarP effector from *Chlamydophila caviae* for which the natural host is guinea pig. TarP is extracellular for the bacterium but the correct cell compartment to use is cytosol for the host cell. The correct species is the host *Cavia porcellus*. In the output, the three recently added VBS motifs (41) are shown as red ovals. All other motif matches are hypothetical.

To use the ELM server correctly for non-Eukaryotic pathogen proteins, the input parameters have to be set up appropriately for the host organism, not for the bacterial species. Figure 3 shows correct settings for the VBS motif-containing effector TarP from *Chlamydophila caviae* that infects the guinea pig (41).

### Cytoskeletal regulatory motifs

**Figure 4.**
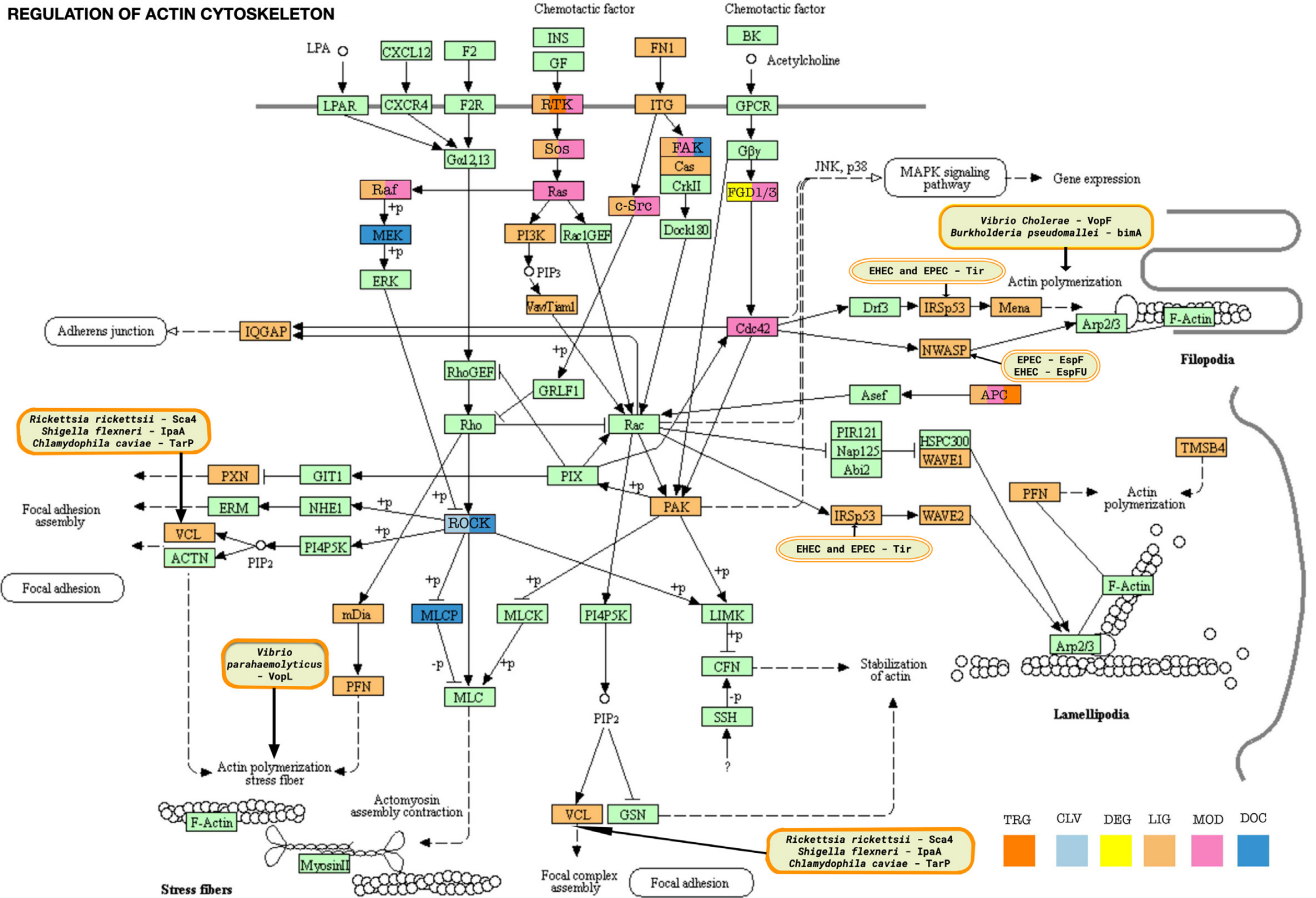
Motif-mediated interactions of the Actin Cytoskeleton network. The KEGG resource network for Regulation of Actin Cytoskeleton (KEGG:hsa04810) is color-coded by ELM motif classes. Proteins of the pathway have a light mint green color by default. Motif-containing proteins are re-colored as follows: DOC class (docking sites) - moderate blue; LIG class (ligand binding motifs) - vivid orange; MOD class (modification sites) - soft pink; DEG class (degradation sites) - yellow; CLV class (cleavage sites) - very soft blue; TRG class (targeting sites) - pure orange; proteins with motifs belonging to multiple classes are marked with the respective colors as described in the bottom right of the figure. ELM has instances for pathogen hijack of actin polymerization at VCL, IRSp53, NWASP and Actin itself. The pathogen proteins affecting these hotspots are shown in the rounded boxes colored with light orange background.

SLiM-mediated interactions play an important role in the control of the actin cytoskeleton, particularly for initiation of actin filament polymerization, and these interactions are often hijacked by bacterial pathogens. Figure 4 shows the KEGG resource (42) Actin Regulatory Pathway color-coded by ELM motif class types and with pathogen intervention sites marked. In the current release of ELM, we have added two new classes (the Profilin-binding polyproline motif and the IRSp53 I-BAR domain-binding NPY motifs) and revised an existing class (Vinculin Binding Sites) that mediate functions associated with the actin cytoskeleton.

Profilin is a key regulator of the cytoskeleton due to its actin-binding and filament-inducing activity. Several actin filament promoting proteins employ poly-proline sequence motifs (New class: LIG_PROFILIN_1) to interact with profilin. Sixteen of these proline-rich motif instances of profilin-binding motifs have been added, including motifs in the key actin regulators WASF1 and VASP.

The I-BAR domain of IRSp53/IRTKS binds NPY motifs (New class: LIG_IBAR_NPY_1) (43–46). The NPY motif was originally discovered in a bacterial pathogenic effector and cellular proteins containing the motif were predicted. The bacterial effector protein Tir of enterohemorrhagic *Escherichia coli* (EHEC) binds IRSp53 with an NPY motif (47,48) to ultimately achieve the activation of actin polymerization and actin pedestal formation. Six new instances including four human motifs and the examples of bacterial IRSp53 hijacking have been added: however, the human examples are all hypothetical motif matches that are plausible but have yet to be validated.

Finally, the Vinculin binding sites class (Revised class: LIG_Vh1_VBS_1) has been updated with a revised regular expression enabling inclusion of several additional instances. Vinculin primarily works as a linker that strengthens the association of Talin and F-Actin at sites of integrin activation, allowing stronger actin binding and stabilization of the sites of focal adhesion (49). Talin contains a long tail with several Vinculin binding sites (VBSs). *Shigella flexneri*, *Rickettsia* and *Chlamydophila* all secrete effectors that mimic Talin VBSs to induce actin polymerization without the need for integrin activation (50–53).

### Membrane-associated pathways

Two novel motif classes involved in membrane trafficking pathways have been added in the current ELM release. A novel class describing a δ-COP interacting motif (New class: LIG_deltaCOP1_diTrp_1) including five new instances has been added. The interaction between tryptophan-based motifs surrounded by negatively charged residues within the lasso-like loop of the Dsl1-tethering complex (54) and the C-terminal μ homology domain (MHD) of δ-COP located in the outermost layer of the coat has an important role in docking COPI vesicles to the ER (55). COPI-coated vesicles mediate the retrograde trafficking pathways from the Golgi to the endoplasmic reticulum (ER) and within the Golgi. The life cycle of COPI-coated vesicles is controlled by essential assembly/disassembly factors, including their specific multisubunit tethering complexes, SNARE complexes and the regulators of their small GTPase Arf1, the ArfGAPs. ArfGAPs (Gcs1p in yeast and ArfGAP1 in mammals) use similar tryptophan-based motifs to interact with the MHD of δ-COP (55).

The classical FFAT motif regular expression has been updated and many new instances have been curated (Revised class: TRG_ER_FFAT_1). A second FFAT class variant with seven instances has also been added to reflect two distinct binding modes (New class: TRG_ER_FFAT_2). FFAT motifs are a class of membrane-protein targeting motifs (56,57), and are important for the formation of membrane contact sites (MCSs) between the ER and cellular membranes (58). The FFAT motifs are recognized by the cytosolic N-terminal MSP domain of the highly conserved VAP integral membrane proteins of the eukaryotic ER. Numerous proteins are targeted to the ER by FFAT motifs and both viral and bacterial pathogens may use FFAT motifs to target the intracellular membrane system of the host. For example, *Chlamydia trachomatis* IncV is a membrane protein on the Chlamydia-containing vacuole, termed the inclusion, that binds host VAP proteins through a FFAT motif (59) to form MCSs that tether the vacuole to the ER.

### Apicomplexan export elements

Apicomplexans are a wide group of unicellular intracellular parasites responsible for various animal and human diseases. *Plasmodium*, *Toxoplasma*, *Cryptosporidium* and *Babesia* are among the most highly studied Apicomplexa genera and they are the parasites that cause malaria, toxoplasmosis, cryptosporidiosis and babesiosis, respectively (60). Apicomplexans invade host cells, remodel them and proliferate inside them, thanks to the coordinated secretion of proteins (61). These proteins are exported using peptide export signals and protein transport complexes, and disrupt the host’s signaling pathways, to sequester nutrients and to evade the immune responses. The Plasmodium Export Element (PEXEL) is the best-characterized export signal in the Apicomplexan phylum. PEXEL is a five residue motif located near the N-terminus of exported proteins following an endoplasmic reticulum (ER) targeting signal peptide (61). It has a dual function: first, as a cleavage site recognized by the aspartyl protease Plasmepsin V and, second, after processing, as a targeting signal to export proteins from the endoplasmic reticulum (ER) through the parasite and parasitophorous vacuole membrane into the infected cell cytosol (61–63). In the current release of ELM, we have added the PEXEL motif as a novel motif class (TRG_Pf-PMV_PEXEL_1). Despite the dual role of the motif, the entry has been added as a targeting motif rather than as a cleavage motif due to its essential role in protein export. We have included 24 novel instances from *Plasmodium falciparum* proteins. These instances are representative of the sequence variation among the PEXELs of other *Plasmodium* species. The regular expression is less strict than the consensus used in the literature, but it should allow the discovery of exported proteins in divergent *Plasmodium* species.

### Expansion of the ELM kinome

In the current release, we present a new motif class describing the modification sites of the DYRK1A kinase (New class: MOD_DYRK1A_RPxSP_1). The dual-specificity tyrosine phosphorylation-regulated kinases (DYRK) family consists of five arginine/proline-directed kinases. The novel motif class describes the specificity of the most studied family member, DYRK1A, which is associated with Alzheimer’s disease, Down syndrome and early onset neurodegeneration (64,65). The optimal DYRK1A phosphorylation site has the consensus R[PSAV].[ST]P motif, however, substrates exist without the consensus proline or arginine and therefore it can act as both a proline-directed and basophilic kinase. The novel DYRK1A class includes 22 motif instances. Since the last ELM release, the modification motif classes of the CK1, CK2 and Cdk kinases have also been revised, expanding the number of instances. In total, 87 novel motif instances have been added to kinase modification site classes.

### Expansion of SH2 motif classes

As a part of the current ELM update, we have significantly expanded the representation of Src homology 2 (SH2) domain binding motifs, grouped under the SH2 functional site. More than 100 SH2 domains are present in mammalian proteomes, where they relay cell state signals through binding to phosphotyrosine motifs that are created following the activation of tyrosine kinases (66). The circa 120 human SH2 domains exhibit a large degree of cross specificity (66,67). Three loops in the SH2 domain determine the accessibility of three hydrophobic pockets, defining clear specificity classes for binding motifs with Asn at position pTyr +2 or hydrophobic residues at positions pTyr +3 and +4 (68,69). We have created three new SH2 classes that reflect their different specificities (New classes: LIG_SH2_CRK, LIG_SH2_NCK_1 and LIG_SH2_STAP1) (40,67,69) and revised an existing class (Revised class: LIG_SH2_GRB2like) (68,70), adding updated structural information to all entries. In total, this has led to the curation of more than 80 individual SH2 motifs and 15 new structures. SH2-binding motifs are not straightforward to annotate as there are many similar preferences revealed by SPOT arrays (66,67). Furthermore, there are examples of peptides that match poorly to the consensus determined by the SPOT arrays but bind with relatively high affinity, perhaps because of the three flexible loops surrounding and contributing to the binding surface (68). Nevertheless, work is ongoing to capture the major SH2 variants in ELM as they are so important in health and disease.

## UPDATES IN THE ELM ANNOTATION PROCESS

SLiM curation is a complex process that requires a curator to read and interpret the relevant information in a motif-related article. New motifs are annotated for the ELM resource by completing two template documents: a text document to describe the motif class and a spreadsheet to annotate instances of a motif class. Both template documents can be downloaded from the ELM website (http://elm.eu.org/downloads/elm_template.doc and http://elm.eu.org/downloads/elm_template.xls). Typically, an annotator will alternate between reading the experimental literature, the motif class template and the motif instances spreadsheet while annotating a new SLiM. We have updated the curation process to simplify annotation activities. We have also improved the motif instance spreadsheet to provide a better overview of the information needed to annotate a SLiM. Furthermore, we have recently prepared a detailed step-by-step protocol on how annotators should work with these templates (Gouw, M. *et al.* (2020) Methods in Mol. Biol., in press). This protocol will serve as a useful guideline for annotators contributing data to ELM, and perhaps even encourage contributions from the research community. The protocol may also be used by developers of other resources to create related guidelines.

## COLLECTION OF PAPERS FOR FUTURE CURATION

The curation of a motif class entry for the ELM resource is a time-consuming process, often taking over a month to complete. This difficulty means that the data in ELM is not comprehensive with regard to motif publications. However, over the past decade, ELM curation has collected over 6000 articles related to SLiMs that await curation, including numerous articles describing novel motif classes. To bridge the gap between the motifs curated in the ELM resource and those awaiting curation, we have created a companion for the ELM resource called articles.ELM. The articles.ELM resource is a literature repository that contains a manually collected compendium of SLiM-related articles. The articles.ELM resource uses text-mining approaches to link novel uncurated articles with motif classes in the ELM resource. This permits a researcher to rapidly find motif literature related to their interests that awaits curation. The resource also allows the deposition of novel articles describing motif data, which are expected to be massively abundant in the upcoming years. The articles.ELM resource is available at http://slim.icr.ac.uk/articles/ and classified articles for an ELM class are available as a link from the ELM class entry page (http://elm.eu.org/elms). For example, the link from DEG_APCC_DBOX_1 (http://slim.icr.ac.uk/articles/browse/?motif_class=DEG_APCC_DBOX_1) returns a total of 152 articles of which 18 are curated in ELM.

## WORKING WITH LINEAR MOTIFS

Reported SLiM instances that are not considered valid are annotated in ELM as False Positives. Most commonly, this is because the suggested motif is buried in the protein fold but sometimes because the interacting protein actually works in a different cellular location. Unfortunately, new examples of False Positive motifs continue to be reported regularly. It is essential to undertake contextual analysis when preparing to investigate a new motif candidate. We have provided guidance to help researchers avoid pitfalls (15). A core set of computational tools that we ourselves use all the time include IUPred, MobiDB and DisProt for assessing intrinsically disordered polypeptide (71–73), JalView and ProViz for motif conservation plus the testing and refinement of Regular Expressions (74,75) and SLiMSearch for searching proteomes (76).

## CONCLUSIONS AND PERSPECTIVES

ELM is a fundamental source of information for the dynamically developing motif biology field. The ELM database is the major resource of quality information on motif-mediated interactions and, thanks to the effort of the motif community, ELM has been continuously developed for almost 20 years. SLiM-mediated interactions constitute a significant and growing fraction of cellular protein–protein interactions (4). They are implicated in diverse human diseases (8,9) and often hijacked by viral, bacterial and eukaryotic pathogens (10–12,62). Therefore, their discovery and characterization is crucial to our understanding of both the physiological and disease states of the cell. We are committed to maintaining, improving and expanding the ELM resource in the future. A key goal for ELM in the coming years will be the addition of new tools to help researchers deal with the anticipated imminent explosion of motif biology information. As ELM approaches its third decade, we believe the resource will continue to support researchers elucidating the key role of motifs in cell biology.

## Notes

Present address: Marc Gouw, Intomics, Lottenborgvej 26, DK-2800 Lyngby (Copenhagen), Denmark.
